# The Swedish version of the Normalization Process Theory Measure S-NoMAD: translation, adaptation, and pilot testing

**DOI:** 10.1186/s13012-018-0835-5

**Published:** 2018-12-04

**Authors:** Marie Elf, Sofi Nordmark, Johan Lyhagen, Inger Lindberg, Tracy Finch, Anna Cristina Åberg

**Affiliations:** 1 School of Education, Health and Social Studies, Dalarna University, Falun, Sweden; 2 Department of Neurobiology, Care Sciences and Society, Karolinska Institutet, Stockholm, Sweden; 3 Department of Health Sciences, Luleå University of Technology, Luleå, Sweden; 4 Department of Statistics, Uppsala University, Uppsala, Sweden; 5 Department of Nursing, Midwifery & Health, Faculty of Health & Life Sciences, Northumbria University, Newcastle upon Tyne, UK; 6 Department of Public Health and Caring Sciences/Geriatrics, Uppsala University, Uppsala, Sweden

**Keywords:** Normalization process theory, NPT, Implementation, Questionnaire, Instrument development, Psychometric properties, Pilot study, Validation, Content validity index

## Abstract

**Background:**

The original British instrument the Normalization Process Theory Measure (NoMAD) is based on the four core constructs of the Normalization Process Theory: Coherence, Cognitive Participation, Collective Action, and Reflexive Monitoring. They represent ways of thinking about implementation and are focused on how interventions can become part of everyday practice.

**Aim:**

To translate and adapt the original NoMAD into the Swedish version S-NoMAD and to evaluate its psychometric properties based on a pilot test in a health care context including in-hospital, primary, and community care contexts.

**Methods:**

A systematic approach with a four-step process was utilized, including forward and backward translation and expert reviews for the test and improvement of content validity of the S-NoMAD in different stages of development. The final S-NoMAD version was then used for process evaluation in a pilot study aimed at the implementation of a new working method for individualized care planning. The pilot was executed in two hospitals, four health care centres, and two municipalities in a region in northern Sweden. The S-NoMAD pilot results were analysed for validity using confirmatory factor analysis, i.e. a one-factor model fitted for each of the four constructs of the S-NoMAD. Cronbach’s alpha was used to ascertain the internal consistency reliability.

**Results:**

In the pilot, S-NoMAD data were collected from 144 individuals who were different health care professionals or managers. The initial factor analysis model showed good fit for two of the constructs (Coherence and Cognitive Participation) and unsatisfactory fit for the remaining two (Collective Action and Reflexive Monitoring) based on three items. Deleting those items from the model yielded a good fit and good internal consistency (alphas between 0.78 and 0.83). However, the estimation of correlations between the factors showed that the factor Reflexive Monitoring was highly correlated (around 0.9) with the factors Coherence and Collective Action.

**Conclusions:**

The results show initial satisfactory psychometric properties for the translation and first validation of the S-NoMAD. However, development of a highly valid and reliable instrument is an iterative process, requiring more extensive validation in various settings and populations. Thus, in order to establish the validity and reliability of the S-NoMAD, additional psychometric testing is needed.

## Background

Implementing new evidence-based interventions, technologies, and ways of organizing health care, with the purpose of improving clinical outcomes and patient experiences, is a complex challenge [1]. If the implementation is not well executed, there is a risk that money and other resources will be wasted due to no or few real sustainable improvements being made. Therefore, in the field of implementation research, aiming to understand what, why, and how interventions work and to test approaches to improve them is urgent [2]. Additionally, the importance of establishing theoretical bases for such research to (i) describe and guide, (ii) understand and explain, and (iii) evaluate implementation processes has been emphasized as important [3].

The Normalization Process Theory (NPT) [4] is an established middle range theory [5] that has been categorized as a theory for enhancing the understanding and explanation of specific aspects of implementation [3]. The NPT is based in social theory and provides an aid for structured interpretation of the issues being researched [6]. It provides support for understanding the dynamics of implementing, embedding, and integrating interventions into routine practice, which in this framework is defined as normalization. It can be used as a conceptual tool, primarily for the investigation of the implementation of complex interventions in health care [7].

The NPT is concerned with explaining what work people do—or need to do—with regard to implementing new practices, which is conceptualized in a set of four core constructs or organizing ideas that represent human processes. These four constructs are Coherence, Cognitive Participation, Collective Action, and Reflexive Monitoring (see Table 1). Coherence concerns the sense-making work that people do individually and collectively to operationalize new practices, while Cognitive Participation mirrors the relational work that people do to build and sustain a community of practice. Collective Action is the operational work that people perform to enact a set of practices, and Reflexive Monitoring is the appraisal work people conduct to assess and understand the ways that a new set of practices affect them and others [4, 8]. According to NPT, it is also possible to investigate the probability or potential of a practice to normalize and become a work routine. The normalization potential [9] can be understood by assessing the factors that are known to affect the implementation process in a specific setting and by the readiness of actors in the work of implementing a new practice and accepting it. The NPT has been widely used for qualitative analyses of the implementation of complex interventions in a diverse range of health care contexts, such as care of chronic kidney disease, chronic heart failure, tuberculosis treatment, maternity care, mental health care and e-health, and tele-treatment interventions [5].

**Table 1:** Overview of the constructs of the Normalization Process Theory and NoMAD items by constructs

Construct	Sub-construct	Items
Coherence	Differentiation	I can see how the [intervention] differs from usual ways of working
	Communal specification	Staff in this organisation have a shared understanding of the purpose of this [intervention]
	Individual specification	I understand how the [intervention] affects the nature of my own work
	Internalization	I can see the potential value of the [intervention] for my work
Cognitive Participation	Initiation	There are key people who drive the [intervention] forward and get others involved
	Legitimation	I believe that participating in the [intervention] is a legitimate part of my role
	Enrolment	I am open to working with colleagues in new ways to use the [intervention]
	Activation	I will continue to support the [intervention]
Collective Action	Interactional workability	I can easily integrate the [intervention] into my existing work
	Relational integration	The [intervention] disrupts working relationships
	Relational integration	I have confidence in other people’s ability to use the [intervention]
	Skill set workability	Work is assigned to those with skills appropriate to the [intervention]
	Skill set workability	Sufficient training is provided to enable staff to use the [intervention]
	Contextual Integration	Sufficient resources are available to support the [intervention]
	Contextual integration	Management adequately support the [intervention]
Reflexive Monitoring	Systemization	I am aware of reports about the effects of the [intervention]
	Communal appraisal	The staff agree that the [intervention] is worthwhile
	Individual appraisal	I value the effects the [intervention] has had on my work
	Reconfiguration	Feedback about the [intervention] can be used to improve it in the future
	Reconfiguration	I can modify how I work with the [intervention]

The growing interest for implementation research has also brought about the development and validation of an increasing number of instruments for measuring implementation activity and progress from different theoretical perspectives [10]. Martinez et al. advocate and provide guidance for the careful development and reporting of work to develop instruments for use in implementation science, in order to advance work in the field. So far, a limited amount of studies have developed NPT-based quantitative approaches [11]. The Normalization Process Theory Measure (NoMAD) is one of the first instruments for measuring implementation from a NPT perspective [8, 12]. The NoMAD is a 23-item instrument used for assessing implementation processes, which reflect the constructs of NPT (Table 1) and provide possibilities for adaptations for specific contexts and study protocols [13]. It is aimed to be a sophisticated, yet simple to administrate, NPT-based assessment tool [14] and is therefore anticipated to be potentially useful in a Swedish context.

The current study presents the processes of translation, adaptation, and pilot testing NoMAD to make it available for use in Sweden. It is a Swedish version of this instrument, which we have named S-NoMAD. In addition, we aimed at creating a digital version of S-NoMAD to make it easy to adapt for use in different contexts. The objectives were therefore to (1) translate the original (UK) version of NoMAD for use in the Swedish context and (2) undertake initial psychometric testing of the instrument in terms of reliability and validity, across a sample of staff involved in the implementation of co-ordinated care planning across health and social services. In doing so, the proposition that a Swedish-translated version of NoMAD can adequately assess the NPT constructs of Coherence, Cognitive Participation, Collective Action, and Reflexive Monitoring is tested.

## Methods

We utilized a systematic approach with a four-step process, including forward and backward translation and expert reviews for the test and improvement of content validity of the S-NoMAD in different stages of development. The final S-NoMAD version was then used for evaluation in a pilot study aimed at implementation of a new working method for individualized care planning. The S-NoMAD pilot results were analysed for internal construct validity and internal consistency, in large following the same pattern for analysis of psychometrical properties as performed by the developer for the original NoMAD [12, 14].

### Description of original development of NoMAD

The original NoMAD [12, 14, 15] instrument was developed using a mixed-methods approach and iterative processes. Instrument development work focused primarily on the research team members collectively generating and testing potential items to reflect each of the four constructs of NPT (Coherence, Cognitive Participation, Collective Action, and Reflexive Monitoring). An iterative process of instrument development was undertaken using the following methods: theoretical elaboration, item generation and item reduction (team workshops), item appraisal (QAS-99), cognitive testing with complex intervention teams (*n* = 23 professional interviewees external to the research team), theory re-validation with NPT experts (*n* = 23 key authors of studies applying NPT), and pilot testing of the instrument (members of a team implementing a shared decision-making tool in secondary care). A version of NoMAD containing 43 NPT construct items was tested in the main validation study, in which online and paper-based surveys were conducted with professional staff implementing a range of health-related interventions, across six different intervention projects. From a total pooled sample of 831 submitted surveys, 522 participants (63%) responded to one or more of the 43 NoMAD construct items, and this represented the dataset for further analysis. Descriptive analysis and consensus methods were used to remove redundancy, reducing the final tool to 23 items (20 NPT construct-specific items plus three general assessment items), the instrument on which S-NoMAD is based.

#### The structure and scoring of the original NoMAD instrument

The NoMAD is divided into three sections. It begins with section A consisting of two questions about the respondent, followed by section B with three general questions about the intervention. Section C) contains 20 specific questions about the intervention, corresponding to the four constructs of NPT (Table 1), with Coherence and Cognitive Participation having four items each, seven items for Collective Action, and five items for Reflexive Monitoring.

The Items in section B are answered on a 10-point Likert scale ranging from ‘Not at all’ to ‘Completely’. The items in part C are answered using a 5-point Likert scale, ranging from ‘Disagree Strongly’ to ‘Agree Strongly’. ‘Neutral’ and ‘Not applicable’ were also given as options to describe respondent’s experiences of using the intervention in the work place [8].

The NoMAD is constructed to provide a flexible ‘bank of items’ [14], with an openness for extensive adaptations concerning which items to use and the wording of the items, for example, to provide more anticipatory assessments. Guidance for how NoMAD can be used and adapted is provided on the website [8]. Based on this position, the originators of NoMAD suggest that the instrument should be viewed as a ‘pragmatic measure’ of implementation [16, 17] and encourage adaptation and flexible application by researchers to their own implementation research and practice needs. Therefore, the NoMAD is presented as four sets of construct items, with reliability and validity data, and does not offer specific instructions for scoring or creating construct measures, which must be used in every study. It is advised that, where assessments at the level of the four NPT constructs are of interest, items within the construct may be averaged to create ‘scores’ that may be compared between constructs, groups, or sites, if appropriate to the purpose of the study. In larger studies, users of NoMAD are encouraged to undertake their own psychometric testing if adaptation or selection of items has taken place for new studies using the NoMAD instrument [14].

In the current study on the development of the Swedish version S-NoMAD, we, in close consultation with the developers of the original NoMAD, made a great effort to follow strictly the original NoMAD in the translation and adaptation processes. In line with this, only a limited amount of adaptations were made for the S-NoMAD use in the following pilot testing (see below). No sum of scores or cutoff values were calculated for the interpretation of the results.

### The translation and adaptation process

**Fig. 1:**
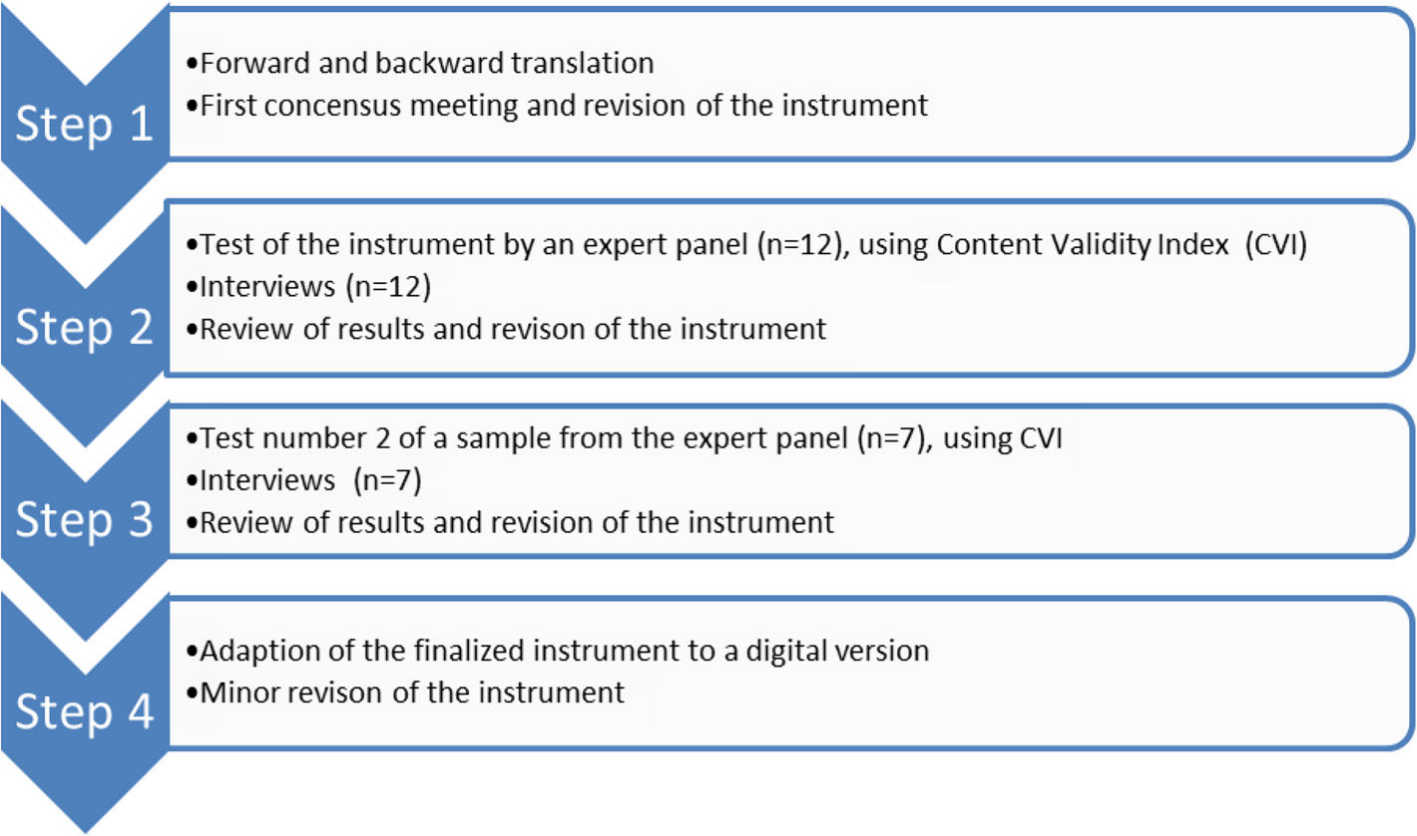
Overview of the translation and adaptation processes

The original version of the NoMAD was received from the developer, and permission was obtained to translate it into Swedish and adapt it to Swedish conditions. In order to ensure conceptual and semantic equivalence between the original and the translated instrument, the approach recommended by Polit and Beck [18] was followed (Fig. 1). This method included forward and backward translations and expert reviews. The content validity and acceptability of the translated NoMAD was assessed in an iterative process with four identified steps, involving (1) forward and backward translation; (2) first test of content validity of the target language instrument, including consultation with experts and further adaptation; (3) final test of content validity of the revised instrument. During the entire translation and adaption process, a number of seminars with researchers were held to discuss the S-NoMAD, and finally (4) adaption of the finalized instrument into a digital version.

#### Step 1: Forward and backward translation

Two of the authors with Swedish as their first language (ME and ACÅ) independently translated the English version of NoMAD into Swedish with the intention of preserving the meaning of each item. The translations were reviewed and discussed before reaching consensus on the most appropriate wording and translation of concepts. The Swedish version of NoMAD was then translated back into English by a bilingual translator with English as a first language. The meaning of the back-translated items and the original items were compared and discussed by ME, ACÅ, and the translator with the aim of reaching satisfactory equivalence between the versions. The developer of the original English version of the instrument (TF) was consulted when needed.

#### Step 2: Test of content validity of the S-NoMAD

A panel (*n* = 12) of researchers and practitioners with experience of being involved in complex health interventions was recruited to participate in the validation of the content of the items in the instrument. Participants were recruited through purposive sampling of researchers and colleagues of the first and last author (ME, ACÅ). The panel consisted of a mix of people in terms of roles and disciplines. The experts were asked to rate all items in terms of relevance using the content validity index (CVI) [19]. In addition, they were encouraged to comment on the items, the expressions of the items, and also the instrument’s form, layout, and legibility.

#### Content validity index (CVI)

CVI is a method used to enhance the construct validity of an instrument. It measures whether the items in an instrument are relevant and the construct is appropriately represented by the items [19]. The items are assessed on a 4-point scale ranging from *not relevant* to *highly relevant*, with an additional response option *do not understand the item*. Item content validity (I-CVI) and scale content validity (S-CVI) were calculated based on the expert ratings. I-CVI was calculated for each item by adding together the number of experts rating the item *quite relevant* or *highly relevant*, divided by the total number of experts rating the item. S-CVI was calculated by summing up the average I-CVI values and dividing them by the number of items.

#### Interviews

Semi-structured interviews were conducted with the 12 experts on the panel. Each interview contained open-ended questions designed to obtain the experts’ reflections on the suitability of the Swedish version of NoMAD for use, and how decisions about the relevance of items had been made. A content analysis on a manifest level was used to analyse the interviews. Two of the authors ME and ACÅ reviewed the results from the experts and identified the items that needed to be revised, i.e. items with I-CVI < 0.78, according to suggestions by Polit and Beck [18]. The experts’ comments also resulted in some modifications being made to the translated version of NoMAD due to semantically vague expressions.

#### Step 3: Test of content on the final adapted instrument

A small number of experts (*n* = 7) once again assessed the content validity of the translated and adapted NoMAD, using CVI. A final revision of the instrument was made.

#### Step 4: Adaption of the finalized instrument to a digital version

Adaption of the finalized instrument to a digital version resulted in minor revisions being made to the instrument. In this step, we worked with a web design firm. The paper-and-pencil and the web-based versions of the S-NoMAD were created to be as similar as possible. The digital version could be used for printouts of paper-based versions. The development of the digital version was performed by two of the authors and a project leader and programmer from the web design firm. The web-based version was discussed in two seminars with a user-group involved in projects that planned to use S-NoMAD. The users were also urged to fill in a document with proposed changes to the instrument.

### The pilot test of S-NoMAD

The first version of S-NoMAD, resulting from step 2 of the translation and adaptation process (Fig. 1), was used in a pilot study that was the starting point of the implementation of the MyPlan intervention. A new Swedish law [20] regulating the process of individualizing care planning in relation to the patients’ discharge from hospital was used as a starting point and to provide framework. The purpose of the MyPlan intervention was to strengthen cooperation between in-hospital care and primary and community care, involving the development and implementation of more flexible and enhanced working methods for health care services. One ambition was to introduce joint meetings between the patient and health service and community care representatives for individualized care planning, documented in a jointly agreed plan, plus a contract establishing the partnership. A new IT solution based on identified patient needs was developed in collaboration with the MyPlan working group, consisting of organizational developers and managers in the involved organizations and two project managers (including one of the authors, SN), and a commercial IT company. A primary goal was to support the improvement of information exchange between professionals working in the county council and community based care.

#### The pilot test of the intervention and data collection using S-NoMAD

The MyPlan pilot study was carried out in two hospitals and four health care centres in two municipalities in a region in northern Sweden. Health care and community care professionals working in these organizations were invited to participate in the implementation of the intervention MyPlan consisting of educational sessions. They had no previous experience of working with the new MyPlan process or the new, related IT system. However, they did have extensive previous experience of working with the former process and IT system (which were replaced by MyPlan), which allowed them to reflect upon and compare obstacles and possibilities between these two approaches during the training session.

S-NoMAD was used to evaluate the normalization potential of the MyPlan concept. For this purpose, context-related adaptations were made concerning (i) specification of professions and roles in relation to the implementation process being initiated and working years and affiliation of participants, (ii) specification of current working methods (which the implementation is aimed at changing), and (iii) adaptation of tense to fit the fact that this first measure by the use of S-NoMAD was carried out in relation to the first stage of the implementation, before the new working method had been practised. If the analysis of the instrument had shown that participants had poor understanding and/or expectations of MyPlan and, hence, that the normalization potential was at risk (which was shown to not be the case), the project management would have had preparedness and time to improve the educational sessions for all participants/staff before the actual implementation.

The pilot study of the intervention MyPlan consisted of a 4-h educational session with information about the new law, new regional collaboration regulation, and new operating procedures supported by the new MyPlan IT system. The sessions were structured to include 2 h of information and discussion and 2 h of practical training using the IT system. This was executed in classrooms equipped with computers, in mixed groups of between 10 and 12 staff members from the concerned organizations. At the end of each session, all 146 health care and community care professionals participating in the pilot study were asked to fill in the S-NoMAD questionnaire. A total of 144 participants (Table 2) completed the S-NoMAD questionnaire, which gave a response rate of 98%. An overview of the pilot study results is shown in Appendix 1.

**Table 2:** Overview of pilot study participants, organization, and work experience

Professions	In-hospital care	Primary care	Community care	Community health care	
Administrator		2			
Occupational therapist	12	1		12	
Home health care organizer			10		
District nurse		6		15	
Head of Unit	2		12	1	
Physiotherapist	13	1		8	
Medical secretary		1			
Registered nurse*	30	6		9	
Assistant nurse	1				
Total	58	17*	22	45	142
					(2 missing)
Professional work experience					
Less than a year	3		4	3	
1–2 years	8	3	10	3	
3–5 years*	7	4	3	4	
6–10 years	9	5	2	5	
11–15 years	5	3		9	
More than 15 years	26	3	3	21	
Total	58	18	22	45	143

*One missing

### Psychometric analysis of the pilot results

A confirmatory factor analysis (CFA) [21] was used to assess the internal construct validity. Due to the small sample size, a one-factor model was fitted for each of the four constructs of the NoMAD based on the NPT. Evaluation of the fit was conducted using standard measures: root mean square of approximation (RMSEA), standardized root mean square residual (SRMR), comparative fit index (CFI), and the Tucker-Lewis index. To determine internal consistency reliability, we used Cronbach’s alpha [22].

## Results

### The translation and adaptation process

There was a high degree of consistency between the backward translation and the original version. Most of the adjustments concerned only precision of language. Some items in the original NoMAD were found to be difficult to translate as they contained words that do not convey the same meaning to Swedes. For example, in the backward translation the word ‘understand’ was used instead of ‘see’, ‘competence’ instead of ‘skills’, and ‘relevant’ instead of ‘legitimate’. How these slight differences in the use of words may change their meaning was discussed with the translator before the final version was decided upon.

The scoring expressions were the most challenging in terms of semantic equivalence. The response options in the original instrument, i.e. strongly agree, agree, neither agree nor disagree, disagree, strongly disagree, could not be used fully. In the Swedish translation, it was difficult to distinguish between the scoring options, due to a translation close to the English wording. We chose to use strongly agree, agree, neither agree nor contradict, contradict, and strongly contradict to make the options more clear. In the pilot test of the instrument, the participants had no queries concerning the wording of the questions of the S-NoMAD questionnaire.

The results of the first content validity analysis showed a S-CVI of 0.84, which is slightly above the recommended level of 0.80, and an I-CVI ranging from 0.5 to 0.92. Four of the items had values less than the critical value of 0.78. The experts thought the items with a low I-CVI were expressed in a difficult and complicated way and that they were difficult to understand because of ambiguous and vague wording. For example, ‘There are key people who drive the intervention forward and get others involved’—this expression addresses two activities in one question, which the experts thought was misleading. Another example of items with low I-CVI was ‘The staff agree that the intervention is worthwhile’. In the first version of the Swedish translation, this had defensive and negative connotations (worth the effort).

A second content validation analysis was carried out on the revised set of items, with I-CVI scores above 0.83 and an S-CVI of 0.96.

The analysis of data from the interviews showed that the majority of the experts welcomed the underlying idea of assessing how well an implementation has been embedded in normal work and using an instrument to do this. Several of the people ran implementation projects and had been looking for a suitable instrument. They welcomed the translated instrument and planned to use it in their future work.

### Psychometric results

#### Internal construct validity

The first factor Coherence consists of four items. Statistical analysis indicates that one factor should be sufficient (*p* = 0.824) and the measures of fit are all acceptable or better indicating that one factor is sufficient with adequate fit. The same applies to the second factor Cognitive Participation consisting of four items (*p* = 0.326), which also has an appropriate fit.

For the third factor Collective Action, which has seven items, one factor is not sufficient (*p* value < 0.01) and the fit measures indicated a bad fitting model. Further analysis indicated that two items were the cause of the bad fit. Removing these two items from the factor model yielded a one-factor model (*p* = 0.339) and good fit. For identification reasons, it is not possible to estimate a factor model where only two items load on one factor. Hence, we did not estimate a two-factor model, but a one-factor model with the remaining items.

For the fourth factor Reflexive Monitoring with five items, we had a similar problem where one item caused rejection of the one-factor model (*p* = 0.027) and bad fit. Discarding this item gave a one-factor model with four items (*p* = 0.873) and good fit.

Most of the factor loadings are between 0.52 and 0.97 when normalizing the factor variances to one (Appendix 2). The exception is one of the loadings for the factor Coherence of 0.39 whereas the other loadings were 0.77, 0.9, and 0.83 respectively. In general, the loadings of the remaining three factors are of a similar size, indicating factor models with no problems of interpretation.

Due to the limited number of observations (*n* = 144), we did not estimate a full four-factor model to be able to estimate the correlations between the latent factors. Instead, a four-factor model with restrictions on the loadings was estimated. The restrictions came from the above estimated one-factor models. The only free parameters were the correlations between the factors. Table 3 displays the estimated correlations. The correlations between the factors are high, or even very high—ranging from 0.356 up to over 0.9.

**Table 3:** Results from analysis of internal construct validity and internal consistency, after exclusion of three items

	*χ* ^2^	*p* value	RMSEA	SRMR	CFI	TLI	Cronbach alpha
Coherence	0.386	0.824	0.000	0.016	1.000	1.007	0.806
Cognitive Participation	2.239	0.326	0.030	0.034	1.000	0.999	0.793
Collective Action	5.674	0.339	0.032	0.039	0.999	0.998	0.831
Reflexive monitoring	0.271	0.873	0.000	0.012	1.000	1.016	0.782

*RMSEA* root mean square of approximation, *SRMR* standardized root mean square residual, *CFI* comparative fit index, *TLI* Tucker-Lewis index

#### Internal consistency

The internal consistency of the four factors and 17 items of the S-NoMAD had Cronbach’s alpha values of above 0.79 (Table 4). An alpha of about 0.8 implies a random error of 0.36, indicating that the factor models yield good reliability.

**Table 4:** Correlation between the constructs (factors) of the Normalization Process Theory

	Coherence	Cognitive Participation	Collective Action	Reflexive Monitoring
Coherence	1			
Cognitive Participation	0.647	1		
Collective Action	0.797	0.356	1	
Reflexive Monitoring	0.920	0.698	0.909	1

## Discussion

The current study presents the translation process, pilot testing, and psychometric analysis of the Swedish version of the original NPT-based British instrument NoMAD [4, 12, 14], known as S-NoMAD. This study contributes to the development, pilot testing, and evaluation of a questionnaire for measuring success in the implementation of complex interventions in health care for use in different Swedish health care contexts. The analysis of construct validity, based on the CFA and goodness-of-fit indices (SRMR, RMSEA, and CFI), showed good fit to the hypothesized model after deleting three items with low internal consistency. These deleted items were ‘The intervention disrupts working relationships’, ‘I have confidence in other people’s ability to use the intervention’, and ‘Feedback about the intervention can be used to improve it in the future’ (see Table 1), which might need to be revised in future revisions of the S-NoMAD. However, the final factor analysis yielded satisfactory factor loadings, suggesting that S-NoMAD reflects the constructs of the NPT [4, 13]. The internal consistency for the four constructs reflected by S-NoMAD (Coherence, Cognitive Participation, Collective Action, and Reflexive Monitoring) in terms of Cronbach’s alpha values ranged from 0.76 to 0.83 and can be considered as indicating good reliability, in concordance with other studies including the results from the still ongoing initial psychometric evaluations of the original NoMAD instrument [14].

The methods used in the present study were chosen with caution to ensure that the outcome should provide psychometric standards that are as credible as possible. The translation methodology used here, including forward and backward translation, has been recommended as a reliable method for translating instruments for research utilization [18]. Additionally, several experts participated in the translation process to secure cross cultural validity of S-NoMAD [23]. We also used two rounds of expert panels and extensive discussions with them and others, at researcher seminars, to ensure that the nuances of the languages were correctly interpreted. The cultural adaptation was performed throughout the entire translation and development process, so it was not considered as being a separate step. This meant that words and expressions were questioned and discussed at all stages of the process until consensus was reached.

Despite the changes that we made (in wording), we consider the adaptation of the original NoMAD to the Swedish version, with its four steps [18] including forward and backward translation, to be carefully and methodically performed and conducted with sensitivity to the original purpose and theoretical foundation of the instrument. Thus, the core of the instrument should remain the same. However, there is no golden standard for instrument translation and adaptation, rather the use of multiple methods, which we applied in our study, is commonly recommended [24]. The CVI methodology used proved to be an important way of visualizing problematic expressions or items, which from the expert panel’s point of view was considered less relevant. It therefore served as a basis for further analysis and discussions with the research team, but was not the sole criteria for item removal or alteration. In combination with the assessment of CVI, we also used interviews with the experts of the panel, which contributed to the adjustment of the Swedish instrument and governed the development process. This enabled interpretations of the reasons why some items got low CVI scoring, which helped us to improve some of them. It is to be noted that the CVI methodology can aid the handling of already existing items, which correspond to the aim of the present study. This, however, is not useful for the generation of other (new) items that might be of importance to adequately measure the underlying construct [24]. On the other hand, the original NoMAD has been tested for relevance earlier in the item generation process [12]. However, in a translation process, the semantic meaning may be lost and a new test of the relevance of the translated instrument is strongly recommended [23].

The very high response rate of over 98% for the pilot test of S-NoMad, which we used for psychometric analyses, is a clear strength. This can be compared to a response rate of > 50% that is commonly viewed as sufficient for most purposes, even though lower response rates are the norm. However, the sample size and population size are also of importance for calculating a sufficient response rate [25]. There was a variation of the questionnaire item non-response with a higher response rate for items in the beginning and lower response rate for items in the last section of the S-NoMAD (see Appendix 1). This variation might be related to the length of the questionnaire, rather than lack of relevance or comprehensibility since the respondents did not express any doubts when filling in the questionnaire [26]. A shorter questionnaire will obviously take less time and effort to complete for the respondents [27] and might be preferable. Our findings on statistically lower performing items, if replicated in other studies reporting the use of NoMAD, can contribute to the future reduction of the item set through further validation. It may also be worth noting that reflexive monitoring items, which appear at the end of the S-NoMAD, are about appraisal of impact and some respondents can find these more difficult to answer. Possible explanations for this may be found in the fact that in the reflexive monitoring section, some of the questions are about future issues such as the provision of resources in the implementation project, which most of the participants in our pilot study did not have a task assignment for nor a possibility to influence. This is supported by the findings of 108 NPT framed studies synthesized by May et al. [11], which revealed Reflexive Monitoring to be the least often applied theoretical construct in the studies. This was because many of the studies reported were feasibility studies, where the impact of monitoring was under-explored. Nevertheless, the respondents in the pilot study gave seemingly adequate responses to the items (see Appendix 1) without associated notable problems.

As mentioned above, the analysis of internal construct validity based on the pilot results and by the use of a CFA [21] indicated a bad fitting model for three of the of S-NoMAD items, leading them to being excluded from the final model. Our interpretations of these results include speculations about cultural language-related differences between expressions in Swedish and in English. For example, the question asking if the intervention disrupts working relationships might be semantically problematic in the Swedish context. The word ‘disruption’ might be too strong in the present context, since Swedish professional relationships are typically built on consensus. The item ‘I have confidence in others’ ability to use the intervention’ also showed a low fit according to the CFA. This might reflect that it can be more demanding to judge others’ ability to execute working tasks than it is to report ideas about one’s own performance. However, the item is relevant since according to NPT [4] and other implementation theories such as the theory of organizational readiness for change [28], the implementation of more substantial changes in health care requires collective actions, reflexions, and peer support to build communal engagement.

Another result that needs to be considered is the partly high correlation between the four factors (representing the NPT constructs), which was unexpected since this has not been shown in earlier studies concerning the original NoMAD, which showed more moderate correlations among constructs [14]. The high correlations between S-NoMAD factors may be related to the relatively small sample size and a data collection performed on only one occasion, in relation to an introduction and before the intervention had been initiated in daily practice. In the present study, the sample size was just above the recommended size in psychometric testing in order to reach a stable co-variation among the items (10 samples per item) [18, 29]. However, the result may also be traced back to the conceptual and semantic equivalence of the translated instrument. For example, the words in the scoring steps used in the pilot test might be too close to each other in order to correctly discriminate the answers (strongly agree, agree, neither agree nor disagree, disagree, strongly disagree). In the translation process, we wanted to stay as close to the original wording as possible, which might have resulted in a translation that semantically differs somewhat from the original language. In a later developed version of S-NoMAD, we slightly changed the scoring expressions of one ‘middle response’ alternative of the scale to improve language clarity, but we did not adjust any endpoints. Given that all of the psychometric tests are relational (and that they all use the same scale) rather than comparative in any absolute sense, we judge that this adjustment of scoring method will only have a very minor influence on the results. A high correlation could also be a sign of item redundancy, which risks diminishing content validity, if the items do not provide one item’s worth of new information related to the NPT construct in question [30]. On the other hand, all the items tapping different attributes of NPT should, therefore, be at least moderately correlated. Otherwise, the homogeneity and internal consistency of the instrument is at risk of being reduced [31]. Considerations concerning appropriate levels of correlations should be allowed to influence the interpretation of the current results and should be analysed again in future revisions and with more extensive tests of the S-NoMAD instrument.

## Conclusions

This article provides access to a quantitative assessment of NPT for research in Sweden known as S-NoMAD, as well as methodological lessons in the development, translation, and testing of much-needed processes and outcome measures for advancing implementation science. It presents the ways in which the NPT-based instrument, NoMAD, was translated and adapted into a Swedish context and the implications for the psychometric stability of the translated version. Our results show satisfactory psychometric properties for the initial step of translation and validation of the S-NoMAD. S-NoMAD is a simple measurement tool that is easy to administrate. As it aims to evaluate how complex interventions are embedded in health care, it could be useful in practice as well as in research and possibly guide implementation processes in ways that will promote normalization. However, the development of a highly valid and reliable instrument is an iterative process, requiring numerous extensive tests and tests in various settings and populations. Thus, in order to establish the validity and reliability of the S-NoMAD, additional psychometric testing is needed.

## Appendix 1

**Table 5:** Overview of descriptive pilot study results (*N* = 144)

English answers alternatives	Strongly agree	Agree	Neither agree nor disagree	Disagree	Strongly disagree	Not relevant to my role	Not relevant at this stage	Not relevant to the intervention	Missing data
Swedish answers alternatives	Instämmer fullständigt	Instämmer	Varken in-stämmer eller motsäger	Håller inte med	Håller verkligen inte med	Ej relevant för min roll	Ej relevant i detta skede	Ej relevant för inter-ventionen	Uppgift saknas
Item	*n* (%)	*n* (%)	*n* (%)	*n* (%)	*n* (%)	*n* (%)	*n* (%)	*n* (%)	*n* (%)
I can see how the [intervention] differs from usual ways of working	17 (11.8%)	99 (68.8%)	23 (16%)	1 (0.7%)	1 (0.7%)	2 (1.4%)	1 (0.7%)	0	0
Staff in this organisation have a shared understanding of the purpose of this [intervention]	6 (4.2%)	42 (29.2%)	65 (45.1%)	17 (11.8%)	5 (3.5%)	5 (3.5%)	4 (2.8%)	0	0
I understand how the [intervention] affects the nature of my own work	13 (9.0%)	59 (41%)	51 (35.4%)	10 (6.9%)	5 (3.5%)	4 (2.8%)	1 (0.7%)	0	1 (0.7%)
I can see the potential value of the [intervention] for my work	14 (9.7%)	67 (46.5%)	43 (29.9%)	11(7.6%)	3 (2.1%)	4 (2.8%)	2 (1.4%)	0	0
There are key people who drive the [intervention] forward and get others involved	22 (15.3%)	82 (56.9%)	31 (21.5%)	4 (2.8%)	0	2 (1.4%)	1 (0.7%)	1 (0.7%)	1 (0.7%)
I believe that participating in the [intervention] is a legitimate part of my role	27 (18.8%)	89 (61.8%)	23 (16%)	1 (0.7%)	0	2 (1.4%)	1 (0.7%)	0	1 (0.7%)
I am open to working with colleagues in new ways to use the [intervention]	44 (30.6%)	89 (61.8%)	8 (5.6%)	0	0	2 (1.4%)	0	0	1 (0.7%)
I will continue to support the [intervention]	40 (27.8%)	87 (60.4%)	14 (9.7%)	0	0	0	1 (0.7%)	0	2 (1.4%)
I can easily integrate the [intervention] into my existing work	9 (6.3%)	38 (26.4%)	64 (44.4%)	20 (13.9)%	8 (5.6%)	3 (2.1%)	1 (0.7%)	0	1 (0.7%)
The [intervention] disrupts working relationships	21 (14.6%)	77 (53.5%)	43 (29.9%)	1 (0 7%)	1 (0.7%)	0	0	0	1 (0.7%)
I have confidence in other people’s ability to use the [intervention]	16 (11.1%)	80 (55.6%)	43 (29.9%)	3 (2.1%)	0		1 (0.7%)		1 (0.7%)
Work is assigned to those with skills appropriate to the [intervention]	12 (8.3%)	67 (46.5%)	54 (37.5%)	9 (6 3%)	0	0	0	0	2 (1.4%)
Sufficient training is provided to enable staff to use the [intervention]	10 (6.9%)	39 (27.1%)	53 (36 8%)	23 (16.0%)	15 (10.4%)	1 (0.7%)	1 (0.7%)	0	2 (1.4%)
Sufficient resources are available to support the [intervention]	9 (6.3%)	33 (22.9%)	64 (44.4%)	21 (14.6%)	15 (10.4%)	0	0	0	2 (1.4%)
Management adequately supports the [intervention]	16 (11.1%)	54 (37.5%)	61 (42.4%)	6 (4.2%)	2 (1.4%)	1 (0.7%)	1 (0.7%)	1 (0.7%)	2 (1.4%)
I am aware of reports about the effects of the [intervention]	6 (4.2%)	46 (31.9%)	60 (41.7%)	19 (13.2%)	4 (2.8%)	2 (1.4%)	2 (1.4%)	0	5 (3.5%)
The staff agree that the [intervention] is worthwhile	10 (6.9%)	43 (29.9%)	70 (48.6%)	10 (6.9%)	1 (0.7%)	2 (1.4%)	4 (2.8%)	0	4 (2.8%)
I value the effects the [intervention] has had on my work	16 (11.1%)	63 (43.8%)	53 (36.8%)	6 (4.2%)	0	1 (0.7%)	1 (0.7%)	0	4 (2.8%)
Feedback about the [intervention] can be used to improve it in the future	44 (30.6)%	79 (54.9%)	16 (11.1%)	0	0	0	1 (0.7%)	0	4 (2.8%)
I can modify how I work with the [intervention]	15 (10.4%)	60 (41.7%)	55 (38.2%)	6 (4 2%)	1 (0.7%)	1 (0.7%)	2 (1.4%)	0	4 (2.8%)

## Appendix 2

**Table 6:** Factor loading and explained variance per item (communality) of the constructs (factors) of the Normalization Process Theory (NPT) constructs, after exclusion of three items (C 3.2, C3.3, and C 4.4)

Items*	Coherence	Cognitive Participation	Collective Action	Reflexive Monitoring	Communality
1.1	0.773				0.598
1.2	0.39				0.152
1.3	0.896				0.803
1.4	0.832				0.692
2.1		0.528			0.279
2.2		0.531			0.282
2.3		0.972			0.944
2.4		0.806			0.650
3.1			0.722		0.521
3.4			0.584		0.341
3.5			0.756		0.572
3.6			0.872		0.761
3.7			0.611		0.373
4.1				0.612	0.375
4.2				0.64	0.410
4.3				0.882	0.777
4.5				0.632	0.399

*Items of section C relating to the four NPT constructs
